# Fox’s Atlas and Text-Book of Skin Diseases

**Published:** 1888-04

**Authors:** 


FOX'S ATLAS AND TEXT-BOOK OF SKIN DISEASES. Second edition. Issued in parts by E. B. Treat & Co., New York. 1888.

Parts three and four of this excellent atlas consider the various kinds of eczema. The plates are wonderfully accurate.

